# Murine Adipose Tissue-Derived Stromal Cell Apoptosis and Susceptibility to Oxidative Stress *In Vitro* Are Regulated by Genetic Background

**DOI:** 10.1371/journal.pone.0061235

**Published:** 2013-04-04

**Authors:** Robert Pazdro, David E. Harrison

**Affiliations:** The Jackson Laboratory, Bar Harbor, Maine, United States of America; University of Tampere, Finland

## Abstract

Adipose tissue-derived stromal cells (ADSCs) are of interest for regenerative medicine as they are isolated easily and can differentiate into multiple cell lineages. Studies of their *in vitro* proliferation, survival, and differentiation are common; however, genetic effects on these phenotypes remain unknown. To test if these phenotypes are genetically regulated, ADSCs were isolated from three genetically diverse inbred mouse strains- C57BL/6J (B6), BALB/cByJ (BALB), and DBA/2J (D2)- in which genetic regulation of hematopoietic stem function is well known. ADSCs from all three strains differentiated into osteogenic and chondrogenic lineages *in vitro*. ADSCs from BALB grew least well *in vitro*, probably due to apoptotic cell death after several days in culture. BALB ADSCs were also the most susceptible to the free radical inducers menadione and H_2_O_2_. ADSCs from the three possible F1 hybrids were employed to further define genetic regulation of ADSC phenotypes. D2, but not B6, alleles stimulated ADSC expansion in BALB cells. In contrast, B6, but not D2, alleles rescued BALB H_2_O_2_ resistance. We conclude that low oxidative stress resistance does not limit BALB ADSC growth *in vitro*, as these phenotypes are genetically regulated independently. In addition, ADSCs from these strains are an appropriate model system to investigate genetic regulation of ADSC apoptosis and stress resistance in future studies. Such investigations are essential to optimize cell expansion and differentiation and thus, potential for regenerative medicine.

## Introduction

Adult stem cells are indispensable for replacing senescent cells and repairing cellular damage, especially in tissues with rapid cell turnover. For example, hematopoietic stem cells (HSCs) maintain the integrity of the hematopoietic and immune systems by constantly replacing component cell types [1], the crypt base cells of the intestine continually renew the epithelial layer of the digestive tract [2], and neural stem cells located in the brain regions of the dentate gyrus in the hippocampus and subventricular zone in the lateral ventricle are required for neurogenesis in adults [3]. Mesenchymal stem cells can be isolated from bone marrow and from the stromal vascular fraction (SVF) of adipose tissue; cells from the latter are termed adipose tissue-derived stromal cells (ADSCs). The *in vivo* role of ADSCs has remained elusive for many years. However, the intravascular location of ADSCs in adipose tissue supports the hypothesis that these cells serve as vascular precursor cells in various stages of development [4].

ADSCs exhibit standard stem cell characteristics, including self-renewal [5] and differentiation into multiple cell types [6] of mesodermal lineages such as osteocytes [7], chondrocytes [8], and adipocytes [9]. ADSCs also may differentiate into non-mesodermal cells, such as neurons [10] and hepatocytes [11]. ADSCs are easily obtained with minimal invasiveness and a large yield; both are significant advantages for potential clinical applications. ADSC-centered treatments are currently being developed for pancreatic regeneration in diabetes [12], [13], [14], therapeutic angiogenesis [15], and differentiation into Schwann cells for nervous system repair [16], [17], [18]. If the potential of ADSCs in regenerative medicine is to be realized, ADSC physiology and regulation must be better understood. Currently, the effects of genetic variation on ADSC function are not defined, but genetic background likely modulates ADSC function since genetic background regulates functions of other stem cell populations like HSCs [19], [20], [21]. Demonstrating that genetic background regulates ADSCs is the first step in elucidating the genetic mechanisms that regulate ADSCs.

Oxidative stress influences ADSCs *in vitro* and may impact their regenerative capacity. Redox alterations and subsequent dysregulation of reactive oxygen species (ROS) production impairs *in vitro* ADSC cell expansion [22]; however, it is unclear if the effects of ROS on ADSC expansion center on stimulation of apoptosis or suppression of proliferation. While oxidative stress appears to affect cell expansion *in vitro*, differentiation often remains unaltered (5). The complexities in the relationship between ROS and stem cell function are highlighted in ischemia-reperfusion injury models, where injection of ADSCs suppresses oxidative stress [23], [24], [25] yet an increase in mitochondrial ROS formation *in vitro* enhances subsequent ADSC-mediated angiogenesis *in vivo* [15]. Because ROS affect ADSC expansion, further study of oxidative stress resistance in these cells is of great importance. The finding that genetic background influences ROS levels in murine HSCs [26] provides further rationale for examining the effects of genetic background on ADSC reactions to oxidative stress.

The present study compares the *in vitro* expansion and oxidative stress resistance of ADSCs isolated from three genetically diverse inbred strains of adult female mice: C57BL/6J (B6), BALB/cByJ (BALB), and DBA/2J (D2) mice. BALB ADSCs had high rates of apoptosis after the initial expansion phase; B6 and D2 ADSCs had significantly lower rates of apoptosis. In F1 hybrids, D2 alleles stimulated BALB ADSC expansion, while B6 alleles did not. BALB ADSCs were also the most sensitive to oxidative stress-induced cell death. In contrast to our findings with cell expansion, B6- but not D2- alleles rescued BALB ADSCs from oxidative stress. Thus, ADSC cell expansion and free radical resistance are regulated by different genes. The antioxidant N-acetyl-cysteine (NAC) did not reduce BALB ADSC apoptosis, confirming that high levels of apoptosis were not due to inadequate antioxidant potential in normal media. Because *in vitro* expansion may be a necessary part of clinical ADSC-based therapies, discovering the effects of genetic background on ADSC cell expansion and stress resistance could lead to the revelation of molecular targets needed to optimize stem cell therapies.

## Materials and Methods

### Materials

Dulbecco's modified Eagle's medium (DMEM), fetal bovine serum (FBS), and penicillin-streptomycin were purchased from Life Technologies (Grand Island, NY). Cell culture ware was purchased from Fisher Scientific (Pittsburgh, PA) and Collagenase A was purchased from Roche (Indianapolis, IN). Dimethylformamide (DMF), thiazolyl blue tetrazolium bromide, H_2_O_2_, menadione, paraquat, and all chemicals for differentiation were purchased from Sigma-Aldrich (St. Louis, MO).

### Mice and ADSC isolation

Female C57BL/6J (B6; JAX^®^ #000664), BALB/cByJ (BALB; JAX^®^ #001026) and DBA/2J (D2; JAX^®^ #000671) mice were produced and housed at The Jackson Laboratory, Bar Harbor, ME, until 4–6 months of age, when they were euthanized by CO_2_ (The Jackson Laboratory Animal Care and Use Committee approved procedure LAH93-26). The same was done for F1 hybrids CByD2F1 (BALB × D2 cross), CByB6F1/J (BALB × B6 cross; JAX^®^ #100009), and B6D2F1/J (B6 × D2 cross; JAX^®^ #100006), except they were euthanized at 2–6 months of age. All studies were conducted under a protocol approved by the Animal Care and Use Committee (ACUC) of The Jackson Laboratory.

ADSCs were isolated from the SVF as previously described [27], [28]. Inguinal fat pads were finely minced and digested in 0.2% collagenase A in PBS. The resultant mixture was passed through a cell strainer (70 µm, Fisher Scientific), and the collagenase was neutralized with growth media (DMEM with 10% FBS and 1% penicillin-streptomycin). The cell suspension was centrifuged for 5 minutes at 1,000 rpm and the supernatant was discarded. After lysis of red blood cells with 1 ml of red blood cell lysis buffer (Sigma-Aldrich), the tubes were centrifuged and the supernatants were again discarded. Cell pellets were washed with PBS and cells were plated on plastic culture dishes in fresh growth media. Non-adherent cells were removed after 4–16 hours; adherent cells were considered ADSCs and were maintained at 37°C and 5% CO_2_. ADSCs were passaged after achieving 80% confluence. To further assure that only adherent cells were used in experiments, ADSCs were grown for two passages before being tested in experiments.

### Distinguishing ADSCs and Fibroblasts

The method we used to isolate ADSCs is well-accepted and does not include a step to exclude fibroblasts for two reasons. First, there are no generally accepted cellular markers that can reliably distinguish between ADSCs and fibroblasts. A recent study by Halfon, et al., showed that markers used to characterize mesenchymal stem cells-including CD44, CD 90, and CD105- are also expressed on fibroblasts [29]. A second reason for not distinguishing between ADSCs and fibroblasts centers on the definition of each cell type. ADSCs and fibroblasts may be much more closely related than previously thought [30], as evidenced by the findings that ADSCs and fibroblasts have similar differentiation capacities [31] and immunomodulatory abilities [32].

### Differentiation

ADSCs were differentiated into osteogenic and chondrogenic lineages as previously described [6], [33]. To test osteogenic differentiation, 2.0×10^5^ ADSCs were plated in each well of a 6-well plate and treated with either growth media or growth media supplemented with 10 mM β-glycerophosphate, 50 µM L-ascorbic acid-2-phosphate, and 10 nM vitamin D3. After 4 weeks, cells were washed with PBS and calcium deposits were stained with 2% alizarin red for 2 minutes followed by extensive washing with DI water. Photos of wells were then captured with a Leica Wild M10 microscope equipped with a Leica DFC300 FX camera.

To test chondrogenic differentiation, ADSCs were plated in 6-well plates at the same density used in osteogenic differentiation experiments. Cells were treated with either growth media or growth media supplemented with 50 µM L-ascorbic acid-2-phosphate, 10^−8^ M dexamethasone, 10 ng/ml TGF-β3, and 1X insulin-transferrin-selenium (Life Technologies). After 4 weeks, sulfated glycosaminoglycans were stained with alcian blue. To do this, ADSCs were fixed inside the wells with 0.5% glutaraldehyde in water, scraped into fixative, and pelleted by centrifugation. The cell pellets were dissolved in warm Histogel (Thermo Scientific), which was allowed to harden into a pellet. The Histogel pellet was processed for paraffin embedding, sectioned, and stained with alcian blue and counterstained with nuclear fast red. Photos were captured using a Leica DMRXE microscope equipped with a Leica DFC300 FX camera.

### General cell expansion and apoptosis

To study cell expansion, ADSCs were seeded at 5.0×10^3^ cells/well in a 24-well plate and allowed to grow for 5, 10, or 15 days. Growth media was changed every 2–3 days. At the predetermined time points, cells were washed with PBS and the amount of double-stranded DNA (dsDNA) was quantified using a *FluoReporter Blue* Fluorometric dsDNA Quantitation kit (Life Technologies), which employs the nucleic acid stain Hoechst 33258. Each individual experiment was analyzed with its own external standard curve that was used to calculate cell numbers per well.

Apoptosis was measured by first plating 2.0×10^5^ ADSCs either in a 100 mm dish for normal growth or in a 6-well plate for confluence. Cells were incubated for 5 or 10 days in culture before being trypsinized and analyzed for apoptosis using an Annexin V-FITC kit (Biolegend, San Diego, CA). Fluorescence ADSC markers were measured with a FACSCalibur flow cytometer (Becton Dickinson, Bedford, MA).

### Immunophenotype analysis

Cell surface characterization was performed by measuring proportions of ADSCs that stained positive for CD44, CD90, and CD105. ADSCs in 100 mm dishes were trypsinized, centrifuged, and washed with PBS. The cells were then stained with the following antibodies tagged with fluorochromes: CD11b-PE-Cy7 (Biolegend), CD44-APC-Cy7 (eBioscience, San Diego, CA), CD45.2-Pacific Orange (JAX Flow Cytometry Service, clone 1042.1), CD90.2-PE (BD Biosciences, San Jose, CA), and CD105-APC (Biolegend). The cells were then analyzed on an LSR II flow cytometer (Becton Dickinson). Percentages of cell populations that stained positive for a given marker were expressed as a percent of live cells, which was determined by propidium iodide (PI) staining.

### Cell viability after oxidative stress

Cell viability was assessed by MTT assay [34]. Briefly, 2.5×10^3^ cells were seeded per well in a 24-well plate and allowed to adhere overnight. Growth media was then replaced with media containing various concentrations of the oxidative stress-inducing agents H_2_O_2_, menadione, or paraquat. After 16–24 hours (depending on the stressor), media was removed and replaced with growth media containing a final concentration of 0.5 mg/ml thiazolyl blue tetrazolium bromide. After 4 hours of incubation at 37°C, cells were lysed using 20% SDS in 50% DMF. After the formazan crystals were fully dissolved, absorbances were read at 570 nm for a test wavelength and 690 nm for background on a SpectraMax 190 microplate reader (Molecular Devices, Sunnyvale, CA). ADSCs from each treatment were run in duplicate or triplicate. The final absorbances were expressed as a percentage of control well absorbance averages.

### Statistics

Results are expressed as mean ± S.E. Statistical significance was determined by ANOVA with a Tukey post hoc test. When comparing two values, Student's t test was used. *P* values<0.05 were considered statistically significant.

## Results

### Differentiation potential

ADSCs are capable of differentiating into osteogenic [7] and chondrogenic lineages [8]. To confirm ADSC differentiation, cells from B6, BALB, and D2 were differentiated for four weeks and evaluated using histological stains; cells incubated in normal growth media for an equal period of time were used as negative controls. After four weeks, osteogenic-differentiated ADSCs and the negative controls were incubated with alizarin red, which stains mineralized matrix red and indicates osteogenic differentiation (Figure S1). ADSCs incubated in normal growth media showed minimal color change, but cells grown in osteogenic media stained red with no apparent strain effect. Thus, ADSCs from all three strains exhibited an osteogenic phenotype after four weeks of differentiation. We also treated B6, BALB, and D2 ADSCs for four weeks in chondrogenic media or normal growth media, which was followed by staining with alcian blue, which stains for sulfated glycosaminoglycans, a marker of chondrogenic differentiation. The strong alcian blue staining of differentiated ADSCs was indicative of chondrogenic differentiation (Figure S1). ADSCs from the three strains had similar abilities to differentiate into osteogenic and chondrogenic lineages.

### Genetic regulation of in vitro expansion

**Figure 1:**
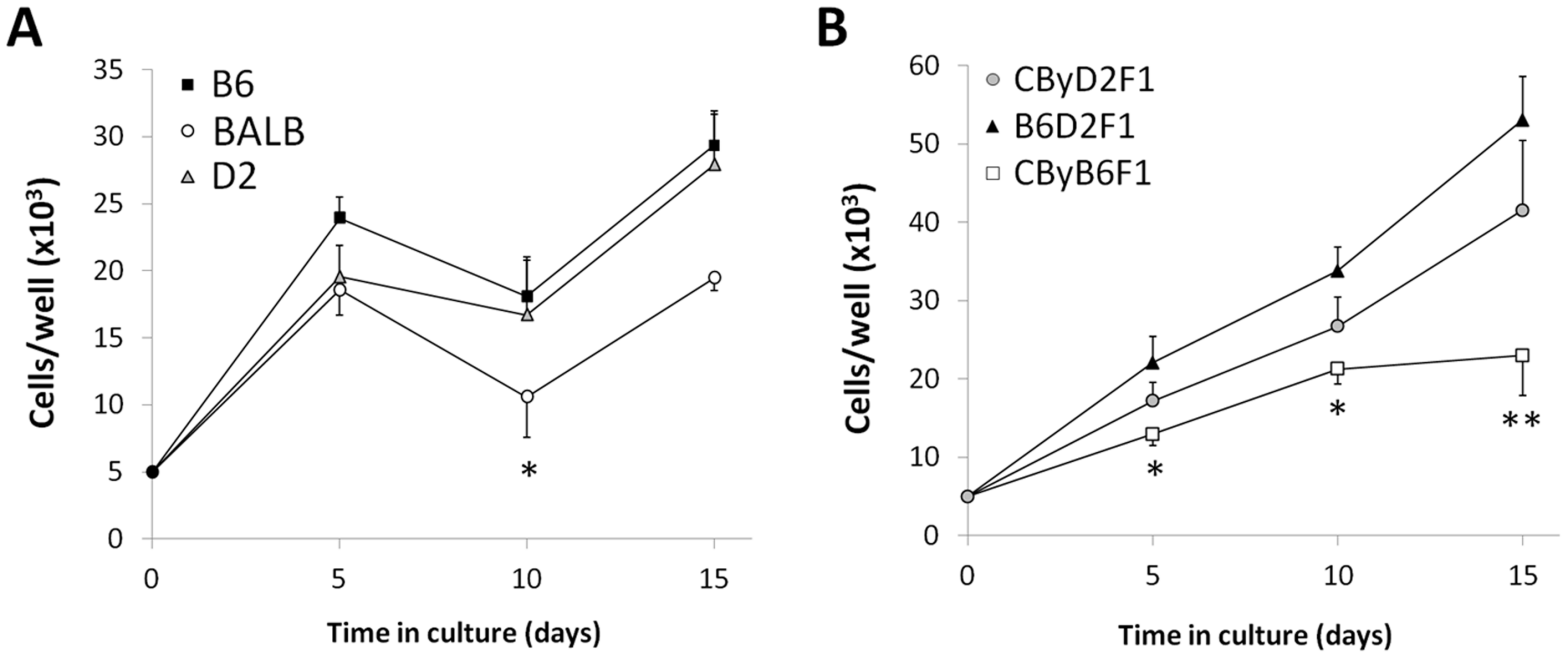
Effects of genetic background on *in vitro* ADSC expansion. ADSCs from B6, BALB, and D2 (A) or their F1 hybrids (B) were plated and grown in culture for 5, 10, or 15 days. Cells were quantified by amounts of DNA tested using Hoechst 33258 and an external standard curve. Data are mean ± S.E. (*n* = 5–8 per data point). In panel A, *P<0.05 vs. BALB ADSCs at day 5 and day 15. In panel B, *P<0.05 and **P<0.01 vs. B6D2F1 ADSCs at respective time points.

To test if *in vitro* ADSC expansion is affected by genetic background, cultured cells were seeded in 24-well plates and allowed to grow for 5–15 days; the number of cells was determined by staining of dsDNA with Hoechst 33258 and calculated using an external standard curve. In all three strains, ADSC numbers increased three to five-fold during the initial 5 days (Figure 1A, Table 1). Between days 5 and 10 in culture, BALB ADSCs significantly declined in number (*P*<0.05 vs. BALB day 5); the numbers of B6 and D2 ADSCs remained steady during this time. The number of BALB cells increased again by day 15 (*P*<0.05 vs. BALB day 10). At 15 days in culture, the numbers of B6 and D2 ADSCs were not different from the number of BALB ADSCs (*P* = 0.09).

**Table 1:** Comparison of ADSC phenotypes between inbred strains and their F1 hybrids.

	B6	BALB	D2	CByD2F1	CByB6F1	B6D2F1
**Cell numbers**						
Day 5	2.4±0.2^a^	1.9±0.2^ab^	2.0±0.2^ab^	1.7±0.1^ab^	1.3±0.2^b^	2.2±0.3^ab^
Day 10	1.8±0.3^bc^	1.1±0.3^c^	1.7±0.4^bc^	2.7±0.1^ab^	2.1±0.2^abc^	3.4±0.3^a^
Day 15	2.9±0.3^bc^	1.9±0.1^c^	2.8±0.4^bc^	4.1±0.2^ab^	2.3±0.5^bc^	5.3±0.6^a^
**Cell viabilities**						
500 µM H_2_O_2_	82±6%^ab^	58±1%^c^	87±3%^ab^	72±4%^bc^	90±3%^a^	90±3%^a^
1000 µM H_2_O_2_	36±13%	6±1%	28±7%	27±7%	42±4%	35±7%

All data given as mean ± SE, with n = 5–7 individual mice, each with 3–5 wells tested to give the mean value for that individual. For cell number, each genotype started with 5.0×10^3^ cells at day 0; data are given ×10^4^. For cell viabilities, all were 100% with no peroxide. Data taken from Figures 1 and 3 and given here to facilitate comparing the six genotypes. All numbers in a row with the same superscript do not differ, while those with different superscripts differ at *P*<0.05.

To further determine the contributions of genetic background in regulating *in vitro* cell growth, we compared growth rates in the following F1 hybrids created from the three parental strains: B6D2F1/J (B6 × D2 cross), CByD2F1 (BALB × D2 cross), and CByB6F1/J (BALB × B6 cross). As with their parental strains, the numbers of F1 hybrid ADSCs per well increased two to five-fold during the first 5 days (Figure 1B, Table 1). By this time, B6D2F1 ADSCs outnumbered CByB6F1 ADSCs (*P*<0.05); this trend continued through days 10 (*P*<0.05) and 15 (*P*<0.01). CByD2F1 cell densities were intermediate and not different from the others; at day 15, the difference between CByD2F1 and CByB6F1 would have reached statistical significance if not for one outlier.

### Genetic regulation of apoptosis

**Figure 2:**
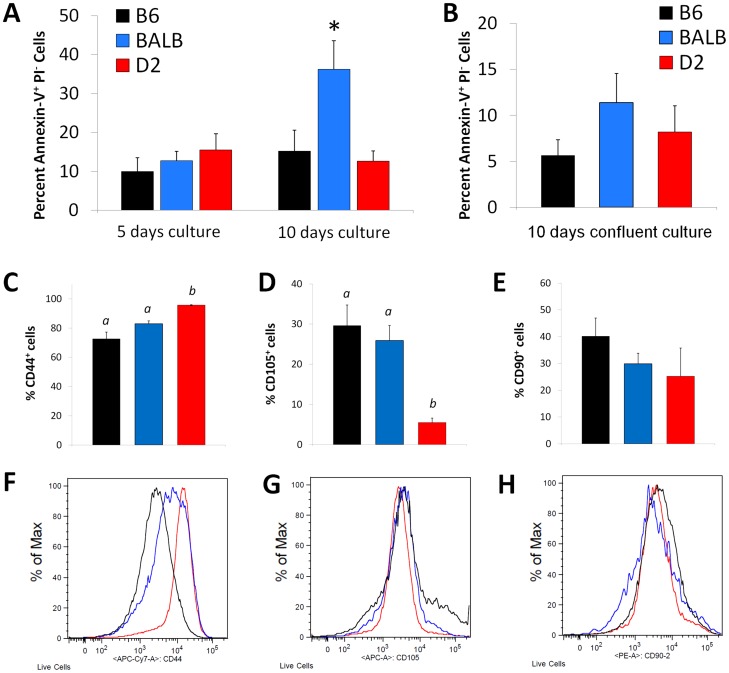
Effects of genetic background on ADSC apoptosis *in vitro*. Apoptosis in B6 (black), BALB (blue), and D2 (red) ADSCs was assessed at 5 days and 10 days in culture; apoptosis was measured by Annexin V-FITC (A). Bar values are expressed as mean ± S.E. (*n* = 5–8). BALB at 10 days differed with *P<0.05 vs. B6 and D2, as well as vs. BALB day 5 and BALB-confluent day 10. B6, BALB, and D2 ADSCs were cultured for 10 days at confluence and apoptosis was assessed (B). Bar values are expressed as mean ± S.E. (*n* = 5–8). To test if cell populations differ by strain, ADSC cell surface markers were analyzed by flow cytometry for the proportion of live cells positive for: CD44 (C, F), CD105 (D, G), and CD90 (E, H). Bar values are mean ± S.E. (*n* = 8–9 per data point). Mean values with dissimilar superscript letters are significantly different (*P*<0.05).

After 10 days in culture, BALB ADSC apoptosis increased significantly compared to the rates at days 5 (*P*<0.05; Figure 2A). BALB ADSC apoptosis rates were also significantly higher than rates in B6 or D2 after 10 days (*P*<0.05 between BALB and B6 or D2); maintenance in dense, confluent cultures suppressed BALB apoptosis (*P*<0.05, Figure 2B). In contrast to the BALB parental strain, the BALB F1 hybrids (CByD2F1, CByB6F1) experienced no decline in cell numbers due to apoptotic cell death; baseline apoptosis (less than 15% of cells Annexin V^+^PI^−^) was observed across all F1 hybrids (data not shown).

We hypothesized that BALB mice may yield a different ADSC population compared to B6 and D2, which could explain its high rates of apoptosis. We used flow cytometry to characterize ADSC immunophenotype with markers typically expressed by cultured human ADSCs: CD44, CD105, and CD90. CD44, which is frequently used to identify ADSCs, was the most commonly expressed marker in all three strains; 72.5±4.7% of B6, 83.0±2.0% of BALB, and 95.7±0.3% of D2 cells were positive for CD44 (Figures 2C and 2F). The proportion of D2 CD44^+^ cells was significantly higher than that observed in B6 and BALB (*P*<0.0001 and *P*<0.05, respectively). Differences between BALB and B6 did not reach statistical significance (*P* = 0.07). In contrast to our findings with CD44, the proportion of D2 CD105^+^ cells (5.5±1.1%) was significantly lower than that of B6 (28.6±5.1%, *P*<0.001) and BALB (25.9±3.7%, *P*<0.005; (Figures 2D and 2G). CD90 expression was not different between strains (Figures 2E and 2H). Taken together, the BALB ADSC immunophenotype is very similar to that of B6 ADSCs, while the D2 ADSC immunophenotype varies significantly from the other two strains in marker profiles. These markers provide no evidence that BALB apoptosis is due to the isolation of a distinct ADSC population that is different from B6. Interestingly, the differences between B6 and D2 populations did not affect function.

### Genetic regulation of oxidative stress resistance

**Figure 3:**
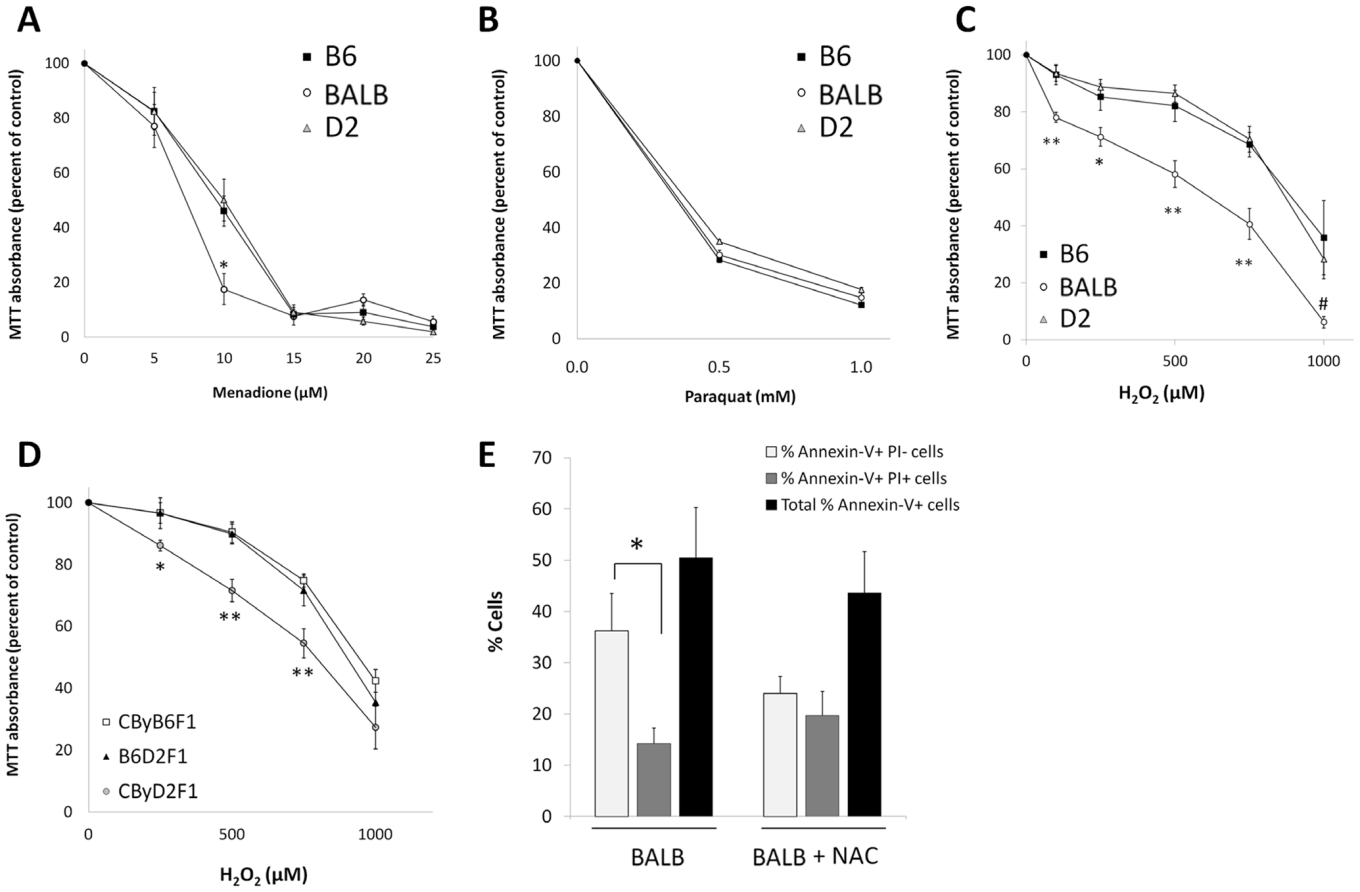
Effects of genetic background on ADSC oxidative stress resistance. MTT assays were performed to assess the cell viability of B6, BALB, and D2 ADSCs following treatment with menadione (A), paraquat (B), and H_2_O_2_ (C). Data are expressed as mean ± S.E. (*n* = 3 for menadione; *n* = 5–6 for H_2_O_2_; *P<0.05 vs. B6 and D2 and **P<0.01 vs. B6 and D2). Cell viabilities of F1 hybrid ADSCs were measured by MTT assay after treatment with H_2_O_2_ (D). Data are expressed as mean ± S.E. (*n* = 5–6; *P<0.05 vs. B6D2F1 and CByB6F1; **P<0.01 vs. B6D2F1 and CByB6F1). BALB ADSCs were treated with and without 4.0 mM NAC in growth media for 10 days and apoptosis was measured (E). Data are expressed as percentages of Annexin-V^+^ cells that are either positive or negative for PI (*n* = 5–7, *P<0.05).

To test if genetic background regulates *in vitro* oxidative stress resistance, we compared cell viabilities after exposing B6, BALB, and D2 ADSCs to the free radical generators menadione (Figure 3A), paraquat (Figure 3B), and H_2_O_2_ (Figure 3C, Table 1). Rates of oxidative stress-induced cell death were strain specific. After 16 hours of incubation with 10 µM menadione, BALB cells were most susceptible to oxidative stress, with 17.6±5.6% viable cells compared to 46.0±5.5% B6 cells and 50.1±7.6% D2 cells (both *P*<0.05 vs. BALB), but there were no differences at other doses of menadione (Figure 3A) or with paraquat (Figure 3B). Cytotoxicity was more pronounced with H_2_O_2_, for which a range of concentrations caused a significantly lower proportion of BALB cells to remain viable compared to B6 and D2 (*P*<0.05 vs. BALB at 100–750 µM H_2_O_2_; Figure 3B). Thus, susceptibility of ADSCs to oxidative stress is regulated by genetic background.

When crossed with D2, but not with B6, BALB ADSCs exhibited increased expansion. Thus, D2 alleles stimulated expansion, while B6 alleles did not. We tested if the trends observed with expansion could be extended to H_2_O_2_ susceptibility, especially because BALB was the inferior strain in both measurements. We measured cell viability following H_2_O_2_ treatment in the three F1 hybrids Figure 3D, Table 1). Like our cell expansion findings, the B6D2F1 ADSCs were the most robust. However, in contrast to our expansion data, the B6- and not D2- alleles rescued the susceptibility of BALB ADSCs to oxidative stress. There was no significant difference between the viability of cells from B6D2F1 mice and CByB6F1 mice following H_2_O_2_ treatment; the viability of CByD2F1 ADSCs was significantly lower than that of the other two F1 hybrids (*P*<0.01 at 500 µM H_2_O_2_ and *P*<0.05 at 750 µM H_2_O_2_). We tested whether the antioxidant NAC- which partially suppressed H_2_O_2_-induced cytotoxicity in preliminary studies- could inhibit BALB ADSC apoptosis Figure 3E). While NAC appeared to influence the relative proportions of BALB ADSCs that were Annexin V-FITC^+^ PI^−^ and Annexin V-FITC^+^ PI^+^, it had no effect on the total percentage of Annexin V^+^ cells, thus confirming that BALB ADSC apoptosis was not due to poor endogenous antioxidant defense. Thus, we conclude that BALB ADSC sensitivity to oxidative stress and high apoptosis in culture are not caused by the same genetic factors.

## Discussion

Ongoing research efforts in many labs are aimed at identifying how ADSCs self-renew and differentiate into cells of diverse lineages. Our studies here demonstrate the importance of genetic background on ADSCs and their *in vitro* characteristics. We began this study by characterizing ADSCs isolated from three inbred strains of mice previously used for adult stem cell research: B6, BALB, and D2. We measured the effects of genetic background on ADSC expansion and apoptosis *in vitro*. While no differences emerged in general cell expansion, we observed that BALB ADSCs undergo high rates of apoptosis while B6 and D2 cells do not. Because we did not observe significant cell death with confluent BALB ADSCs during the differentiation experiments, we also studied ADSCs that had been seeded at confluent densities. Confluence suppressed BALB ADSC apoptosis, suggesting that BALB apoptosis is related to the growth phase and lack of cell-cell contact [35]. We performed immunophenotype analysis on ADSCs from all three strains to test if expression levels of ADSC markers are associated with the high rates of BALB ADSC apoptosis. Expression of CD44, CD90, and CD105 did not differ between cells from BALB and B6, suggesting that BALB ADSC apoptosis is likely not due to strain effects on ADSC markers; differences between D2 and B6 did not affect *in vitro* cell characteristics.

We next examined the expansion of ADSCs from F1 hybrids. B6D2F1 ADSCs grew efficiently, which highlighted hybrid vigor, as their growth rates almost doubled those of ADSCs from either parental inbred strain. The dominance of D2- and not B6- genetic alleles over those of BALB were evidenced by the greater growth patterns of both B6D2F1 and CByD2F1 ADSCs over cells from CByB6F1. We conclude that genetic background influences the expansion of mouse ADSC; furthermore, D2 alleles- and not B6 alleles- were able to stimulate BALB ADSC expansion, suggesting that cell growth exhibits a hierarchal pattern of dominance: D2 > BALB > B6.

Because the effects of genetic background on ADSC oxidative stress resistance are unknown, and because cell culture conditions often encourage free radical production [36], we tested if the genetic background regulates ADSC free radical susceptibility. BALB ADSCs were more sensitive to the free radical generators H_2_O_2_ and menadione compared to B6 or D2 ADSCs; however, there was no exceptional BALB sensitivity to paraquat. We hypothesize that this occurred because of different mechanisms of toxicity. H_2_O_2_ is itself an ROS that can be directly taken up by cells; we used concentrations up to 1000 µM, which previous studies confirm are cytotoxic *in vitro* [37]. However, menadione and paraquat both require intracellular metabolism to generate ROS. Paraquat is reduced at the mitochondria [38], creating a paraquat cation radical that can reduce molecular oxygen (O_2_) to a superoxide radical (O_2_•^−^), which is then converted to H_2_O_2_ by superoxide dismutase. Reduction of O_2_ regenerates the parent paraquat molecule and enables redox cycling mechanisms. Menadione undergoes similar mechanisms of redox cycling [39] that induces mitochondrial damage and cell death [40]. The requirement for intracellular metabolism of menadione and paraquat to induce oxidative stress appears to diminish the strain differences observed with direct administration of H_2_O_2_.

After verifying that H_2_O_2_ increased intracellular ROS (data not shown), we tested the effects of H_2_O_2_ on F1 ADSC viability. As we found with cell expansion, B6D2F1 ADSCs were the most robust in free radical resistance. In contrast to our findings with cell expansion, however, the B6 background- and not D2- rescued BALB susceptibility to the H_2_O_2_ treatment. The disparate capabilities of the D2 and B6 backgrounds to improve BALB ADSC outcome measures show that cell expansion and stress resistance have independent genetic regulation.

The antioxidant NAC partially suppressed H_2_O_2_-induced cell death in preliminary experiments, so we tested whether NAC could inhibit BALB ADSC apoptosis in culture. NAC appeared to influence the proportion of cells that were actively apoptotic from those that had completed apoptosis, but it had no effect on the overall percentage of apoptotic BALB cells. This result supports the idea that the low growth rates and high apoptosis of BALB ADSCs *in vitro* are not due to the impaired free radical resistance.

These data illustrate the importance of genetic background on ADSC function and highlight similarities with another population of adult stem cells: HSCs. Consistent with previous HSC research findings [19], [20], [21], we observed that BALB ADSCs are more sensitive to stress than cells from B6. BALB ADSCs were prone to increased apoptosis and higher susceptibility to oxidative stress compared to ADSCs from B6 and D2. Of course, HSC functions are tested *in vivo*, and it remains unknown whether the BALB deficiencies in growth and stress resistance will affect function and regenerative capacity *in vivo*. We will follow up on the studies presented in this paper in an effort to identify the loci and genes responsible for genetic regulation of ADSCs, both *in vivo* and *in vitro*, which will enable us to test whether some of the same genes regulate ADSCs and HSCs.

Eventual clinical use of ADSCs is expected to center on autologous transplants of ADSCs. Thus, a patient requiring such treatment will be likely to receive his or her own cells. To generate a sufficient amount of cells for successful clinical therapy, ADSCs may require *in vitro* expansion before injection back into patients. Our data show that genetic factors contribute to *in vitro* apoptosis of cultured murine ADSCs and suggest that genetic variations may affect the expansion of human ADSCs. Defining genetic regulators of cell expansion may highlight methods to improve ADSC function, which may be particularly important if it becomes possible to receive cells from genetically dissimilar individuals. Because ADSCs lack expression of HLA-DR, clinical benefit could potentially be achieved following injection of cells from an unmatched donor [41], [42]. Our study also showed that free radical resistance is affected by genetics, but in a manner different from that of *in vitro* expansion. Thus, definition of genetic regulators of stress resistance may be vital for ADSC therapy, especially in conditions with an oxidative stress component, such as diabetes, neurodegenerative disease, and ischemia-reperfusion injury.

## Supporting Information

Figure S1 **B6, BALB, and D2 ADSCs were cultured and differentiated for 4 weeks into osteogenic or chondrogenic lineages.** Cells grown in normal growth media for that time were used as negative controls. Cells were stained using either alizarin red (osteogenic) or alcian blue/nuclear fast red (chondrogenic, as specified by arrows). Pictures were captured using a Leica microscope and are representative of three independent experiments.
(PDF)
