# Supplementary material for: Murine Adipose Tissue-Derived Stromal Cell Apoptosis and Susceptibility to Oxidative Stress In Vitro Are Regulated by Genetic Background
Source: PLoS One. 2013 Apr 4;8(4):e61235. doi: 10.1371/journal.pone.0061235 (PMC3617166; doi:10.1371/journal.pone.0061235)
Supplement: Figure S1 — B6, BALB, and D2 ADSCs were cultured and differentiated for 4 weeks into osteogenic or chondrogenic lineages. Cells grown in normal growth media for that time were used as negative controls. Cells were stained using either alizarin red (osteogenic) or alcian blue/nuclear fast red (chondrogenic, as specified by arrows). Pictures were captured using a Leica microscope and are representative of three independent experiments. (PDF) [file pone.0061235.s001.pdf]

Figure S1. Histological staining of differentiated ADSCs.

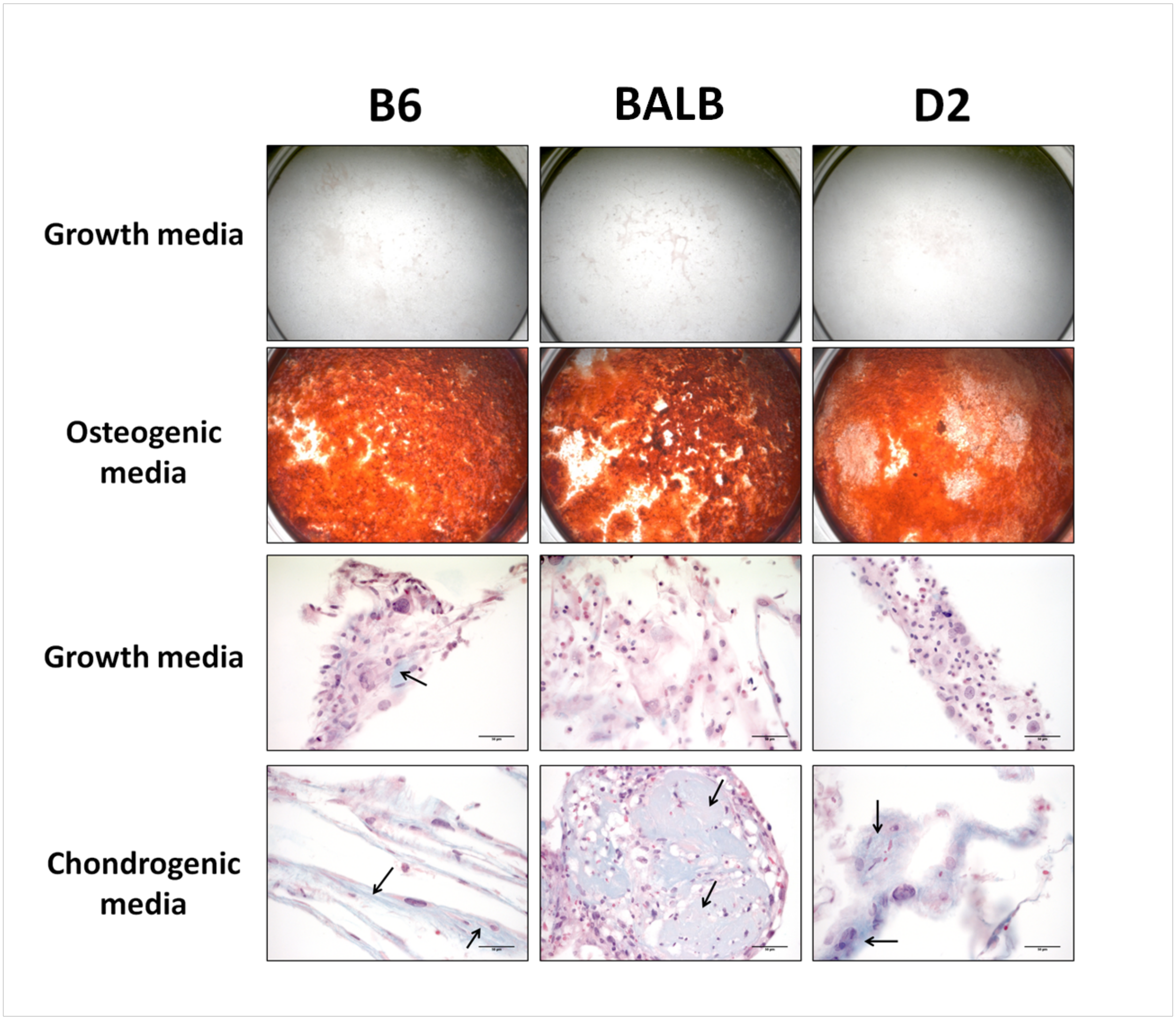

B6, BALB, and D2 ADSCs were cultured and differentiated for 4 weeks into osteogenic or chondrogenic lineages. Cells grown in normal growth media for that time were used as negative controls. Cells were stained using either alizarin red (osteogenic) or alcian blue/nuclear fast red (chondrogenic, as specified by arrows). Pictures were captured using a Leica microscope and are representative of three independent experiments.
